# Numerical Investigation on the Compressive Behavior of Desert Sand-Based Backfill Material: Parametric Study

**DOI:** 10.3390/ma16103878

**Published:** 2023-05-22

**Authors:** Haitian Yan, Honglin Liu, Guodong Li, Xiangyu Wang, Yinjian Hang

**Affiliations:** 1 School of Geology and Mining Engineering, Xinjiang University, Urumqi 830046, China; 107552101622@stu.xju.edu.cn (H.Y.); cklgd2011@xju.edu.cn (G.L.); 2 Key Laboratory of Environmental Protection Mining for Minerals Resources at Universities of Education Department of Xinjiang Uygur Autonomous Region, Xinjiang University, Urumqi 830047, China; 3 School of Mines, China University of Mining and Technology, Xuzhou 221116, China; wangxiangyu79@126.com; 4 Xinjiang Sail Energy Co., Xuzhou Coal Mining Group, Tacheng 834700, China; hangyinjian@126.com

**Keywords:** desert sand, high-water backfill material, PFC3D, parametric study

## Abstract

As a key node in the promotion of the “Western Development” strategy in Xinjiang, China, the large-scale mining of coal resources is bound to cause a series of ecological and environmental problems, such as surface subsidence. Desert areas are widely distributed in Xinjiang, and from the perspective of reserves and sustainable development, it is crucial to fully utilize desert sand to make filling materials and predict its mechanical strength. In order to promote the application of High Water Backfill Material (HWBM) in mining engineering, a modified HWBM doped with Xinjiang Kumutage desert sand was used to prepare a desert sand-based backfill material, and its mechanical properties were tested. The discrete element particle flow software PFC3D is used to construct a three-dimensional numerical model of desert sand-based backfill material. The parameters such as sample sand content, porosity, desert sand particle size distribution, and model size are changed to study their impact on the bearing performance and scale effect of desert sand-based backfill materials. The results indicate that a higher content of desert sand can effectively improve the mechanical properties of HWBM specimens. The stress–strain relationship inverted by the numerical model is highly consistent with the measured results of desert sand-based backfill materials. Improving the particle size distribution of desert sand and reducing the porosity of filling materials within a certain range can significantly improve the bearing capacity of desert sand-based backfill materials. The influence of changing the range of microscopic parameters on the compressive strength of desert sand-based backfill materials was analyzed. This study provides a desert sand-based backfill material that meets the requirements of mine filling, and predicts its strength through numerical simulation.

## 1. Introduction

High-water backfill materials (HWBMs) have high early strength, a short gelling time, a simple construction technology, and other advantages [1,2,3]. Many mines have used HWBMs for goaf filling and roadway side support [4,5,6]. However, filling materials with a high-water content are expensive and decrease in strength with the passage of time. With the promotion of gob-side entry retaining technology and the expansion of the application scope of the HWBM in mine engineering [7,8,9], the performance of HWBMs in engineering practice is further improved.

In fact, a modified HWBM has the same mechanical properties as pure high-water backfill material. Many scholars use different materials, such as fly ash, river sludge, silica fume, and polyethylene plastics, to modify high-water backfill materials and analyze the changes in the properties of HWBMs [10,11,12,13,14]. On adding fly ash, the uniaxial compressive strength and the elastic modulus of the HWBM decrease, the cost decreases, and the residual strength increases [15]. The influence of lithium carbonate and aluminum sulfate on the properties of HWBM was analyzed via a hydration heat test, X-ray diffraction, a scanning electron microscope, and other microscopic tests. It was found that the addition of lithium carbonate and aluminum sulfate can promote HWBM hydration, shorten the solidification time, and improve the early and late compressive strength of the hydration [16,17].

However, most of the coal mines in northwest China are located in arid and semi-arid areas that are short of water. Water resources are precious. It is a potential method to improve its performance to make desert sand-based backfill material with abundant desert sand. The use of desert sand as fine aggregate for concrete production has become a hot research issue, and relevant studies have proved that desert sand concrete has good mechanical properties. Reasonable grading of desert sand aggregate can make concrete denser, reduce the water consumption and cement consumption of concrete mixture per unit volume, and also make the skeleton and stability of aggregate reach a good state [18,19,20,21].

The discrete element method was first proposed by Cundall [22], and the PFC particle flow method, as a kind of discrete element method, is mostly used to study the meso-mechanical properties of granular medium materials. Discrete element particle flow software PFC3D is based on the discrete element method DEM code to track the trajectory of each particle. At present, many scholars build discrete element numerical models by different methods. YingYan et al. [23] established the DEM numerical model by using the quaternion method and simulated the direct shear test of irregular limestone gravel. Y.H. et al. [24], combined with the improved bond particle model, considered the physical characteristics of solid bonds when measuring the compressive strength in the unconfined compaction process, proving that the solid bond model must be considered when using the DEM method to simulate the particle compaction process. Ahmadi et al. [25] conducted a numerical study on the scaling method of polygon particles in DEM and calibrated the model according to the shear test. Peña et al. [26] studied the mechanical response of non-viscous particle materials under monotone loading through molecular dynamics simulation. Tanoli et al. [27] used the zoning method to conduct numerical analysis on deep foundation pit engineering in Shanghai, providing a comprehensive solution for predicting the displacement related to deep excavation in soft clay. Numerical simulation of PFC3D can simulate mechanical parameters and failure forms of research objects more accurately [28,29], but most studies mainly focus on the mechanical properties of research objects, and studies using PFC3D to study the microscopic parameters of models are relatively few. Based on previous studies, this study used discrete element particle flow software PFC3D to build a parallel bond model, study the influence of some parameters on desert sand-based backfill materials, and predict its compressive strength.

The main factors affecting the mechanical properties of desert sand-based backfill materials are the properties of desert sand, such as the content of desert sand, particle size distribution of desert sand, etc. [30]. In addition, porosity is also one of the factors affecting the mechanical properties of rocks and building materials [31]. Therefore, in this paper, we mainly use the discrete element particle flow software PFC3D to carry out numerical inversion. By adjusting the range of the mesoscopic parameters, it analyzes the relationship between the mesoscopic parameters and the macro mechanical properties of the desert sand-based backfill material and further discusses the influence of the sand content, porosity, desert sand particle size distribution range, and model size on the compressive strength of the desert sand-based backfill material model. The research results are of great significance to the practical application of desert sand-based filling materials in mining filling.

## 2. Experimental Study

Previous studies have shown that the water–cement ratio and the content of impurities influence HWBMs [32,33]. Guodong Li et al. [34,35] used the HWBM from Yangzhou China Mining Construction New Material Technology Co., Ltd. (Yangzhou, China), selected the water–cement ratio of the HWBM to be 1, 1.5 and 2, set the desert sand doping amount to be 0, 30%, and 60%, and made seven types of desert sand-based filling material samples. Through a series of tests, the influence of desert sand-based backfill material on its initial setting time, mechanical properties, microstructure, and the effect of water–cement ratio on the performance of HWBM was studied.

**Figure 1:**
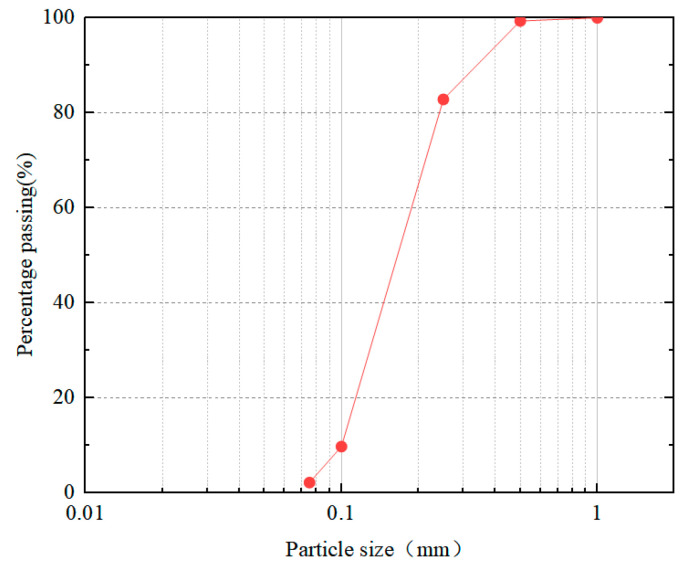
Grain size gradation curve of desert sand.

See Table 1 for specific specimen information, and see Figure 1 for desert sand particle size grading curve. The test piece is numbered according to the different parameters of the test piece, and the test piece is numbered with H. The first number represents the water–cement ratio, and the last number represents the sand content of the test piece. For example, the water–cement ratio is 1, the sand content is 0%, and the test piece number is H-1-00.

The test results show that the initial setting time of the HWBM modified by desert sand is reduced, the high content of desert sand can effectively improve the compressive strength and elastic modulus of the HWBM, the water–cement ratio increases, and the compressive strength of the specimen decreases. By adding desert sand to modify the HWBM, the compressive performance of the test piece has changed. When the sand content of the test piece reaches 60%, its compressive performance has been significantly improved. According to the calculation, the uniaxial compressive strength of specimens H-1-60, H-1.5-60, and H-2-60 increased by 46.63%, 78.04%, and 113.23%, respectively, compared with that without desert sand. Therefore, high content of desert sand can effectively improve the compressive strength of HWBMs. At the micro level, the addition of desert sand changes the microstructure of the HWBM, generates more hydrated calcium silicate (C-S-H) to fill in the gap between ettringite crystals, and its compactness increases, thus improving the mechanical properties of the HWBM.

**Figure 2:**
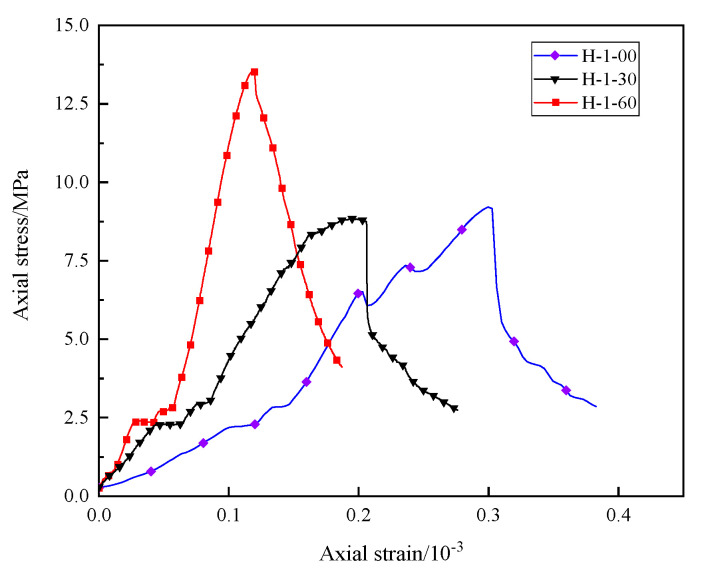
Stress–strain curves of different specimens under uniaxial compression.

Based on the test results, the stress–strain curves of different samples under uniaxial compression when the water–cement ratio is 1 are drawn, as shown in Figure 2. It can be seen that, during compression failure, as the compressive stress increases, the specimen roughly undergoes four deformation stages: (1) In the initial compression stage, under the influence of the compressive stress, the cracks in the specimen gradually close, the stress increases slowly, and the strain increases rapidly, which is the compaction stage. (2) With the increase in compressive stress, the stress and strain increase linearly and uniformly with time, which is a linear elastic failure stage. At this stage, the pores and cracks in the specimen are gradually compacted under the axial stress, and a new fracture surface begins to appear at the same time. (3) When the stress is close to the peak value, the specimen is damaged many times in a short period, and the stress fluctuates many times and reaches the peak value, which is the yield stage. At this stage, the internal structure of the specimen is rapidly destroyed, and the cracks are interconnected to form a macro fracture surface. (4) When the peak stress is reached, the specimen collapses, and the compressive strength rapidly decreases to a lower value, which is the residual stress stage. However, due to the friction between the fracture surfaces, the specimens can still be loaded by friction.

## 3. PFC3D Numerical Model Establishment

### 3.1. Contact Constitutive Model

In PFC3D software, the constitutive model between discrete elements is the basis for realizing the macro mechanical behavior simulation. PFC3D software includes the Hertz contact model, linear contact model, contact bond model, and parallel bond model. Among them, the parallel bond model is used to bond the mechanical behavior of materials, such as the bond of aggregate between cement. Its bonding component is parallel to the linear element and establishes elastic interaction between contacts. The existence of parallel keys does not exclude the possibility of sliding. Parallel bonding can transfer forces and moments between different entities.

**Figure 3:**
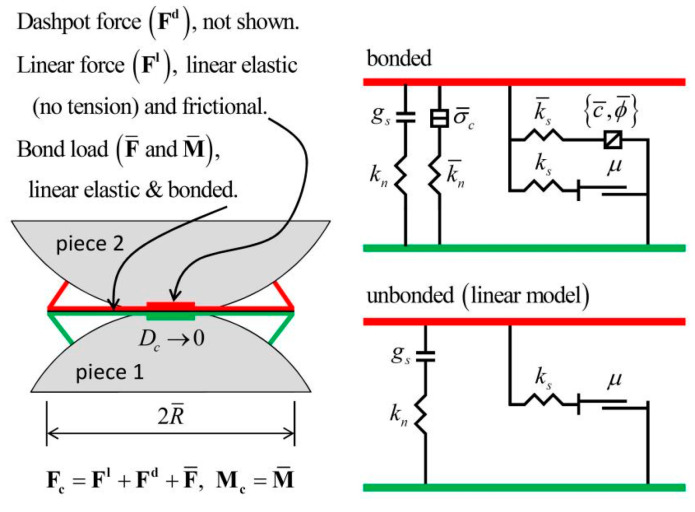
Linear parallel bond model.

The linear parallel bonding model provides the behavior of two interfaces: the friction interface with finite size and the moment bonding interface (Figure 3). The friction interface is equivalent to a linear model: it does not resist relative rotation and adjusts the sliding force by applying the Coulomb limit to the shear force. The moment bonding interface is called a parallel bond because it is parallel to the friction interface during bonding. When the moment bond interface is bonded, it can resist relative rotation, and its behavior is linear elastic until it exceeds the strength limit, and the bond breaks, making it lose its bond. When the second interface is unbound, it will not carry any load. The unbonded linear parallel bonded model is equivalent to the linear model [36].

### 3.2. Model Construction

In this paper, three different proportions of desert sand-based backfill material with a water–cement ratio of 1 are used as the research object. The discrete element particle flow software PFC3D was used for the simulation, and the influence of sand content, porosity, desert sand particle size, and model size on the performance of the test pieces was analyzed. Particle flow analysis usually regards a discrete medium as the aggregate of particle units. Each particle unit moves independently and is in contact with, and interacts with, other particle units. The particles themselves are rigid bodies that can overlap each other but cannot deform. The interaction between particles is realized through different contact models.

We obtained the rock strength, elastic modulus, and other parameters through a uniaxial compression test in the laboratory and conducted the rock uniaxial numerical simulation test using PFC3D. The numerical model adopts the parallel bonding contact model and the linear model. The parallel bonding model is used between the material particles, and the linear model is used between the sphere and the wall. First of all, the domain is created to determine the limits of the area where the model is to be built, and then three walls are set up, which are the loading and download boards of infinite size and the side confining plate, respectively. After that, the particles are generated. First, a region is created using geometry, and then spherical particles are generated in the geometry region by using the ball command. At this point, it will be found that the locations of some particles are beyond the range of geometry and wall; in this case, it is likely to appear that the particles are extruded from the wall, so we can reduce the size of the sphere in the X, Y, and Z directions to make the particles shrink, so as to avoid the bad effect of this situation on the simulation [29].

**Figure 4:**
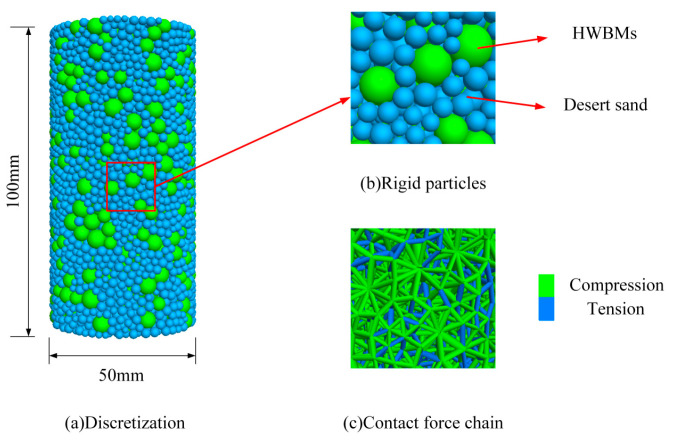
Discrete element modeling desert sand-based backfill material.

The structural system of a desert sand-based backfill material is composed of skeleton particles, and the actual sample particles are in thousands, so a calculation model is difficult to create. To reduce the calculation amount, we adopted the radius amplification method. Thus, the minimum particle radius was set to 1 mm, and the ratio of the maximum radius to the minimum radius was 1.66. The grain unit of the HWBM was randomly generated according to the particle size range of 2~3.32 mm in the designated space according to a uniform distribution, while the grain unit of desert sand was randomly generated according to the particle size range of 1~1.66 mm in the designated space according to a uniform distribution. The size of the model was 50 × 100 mm (width × height), the porosity was 0.3, the density of the HWBM was 1800 kg/m^3^, and the density of desert sand was 1500 kg/m^3^. The generation model is shown in Figure 4, where green particles represent the HWBM and blue particles represent desert sand.

### 3.3. Parameter Calibration

To obtain a macroscopic response consistent with the indoor experimental results in the particle flow program, it is necessary to adjust the microscopic parameters to obtain the macroscopic parameters in line with the actual conditions. When building the PFC3D numerical model, the trial-and-error method is generally used to calibrate the mesoscopic parameters. Adjust the microscopic parameters of particles and bonds multiple times through trial and error until these parameters better reflect the mechanical properties of real rocks [36,37,38,39]. Firstly, the particle flow model is given the estimated mesoscopic parameters, and the numerical simulation results are compared with those of the laboratory tests. If the difference is large, the microparameters of the particle flow model are adjusted continuously by the trial-and-error method, and the model parameters are adjusted many times until the numerical simulation results are basically consistent with the indoor test results.

**Figure 5:**
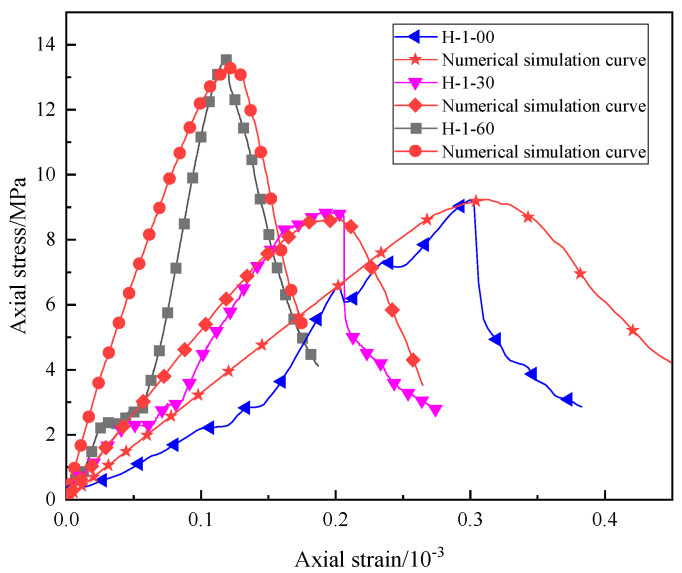
Comparison of stress–strain curves of the laboratory test and the numerical simulation uniaxial compression test.

The specific numerical model test particle mesoscopic parameters are shown in Table 2. The stress–strain curve of the sample calculated using the particle mesoscopic parameters shown in Table 2 is shown in Figure 5. The mechanical parameters and failure patterns of indoor tests and numerical simulations are shown in Table 3. Through comparison, it can be seen that the uniaxial compression stress–strain curve of numerical simulation is relatively close to the uniaxial compression stress–strain curve of the indoor test, and the mechanical parameters of the indoor test and numerical simulation in Table 3 are similar. However, the curves of specimens H-1-00 and H-1-30 after failure are slightly different from the actual situation. The selected particle size is different from the actual particle size of the sand used, so the internal connections and actual cementation are different. This situation can be well simulated before the destruction; after the destruction, it cannot be consistent with reality. The uniaxial compression test is also a process of energy exchange to destroy the desert sand-based backfill material specimens: part of the energy is used for pore crack compaction and crack extension, and the remaining part is stored as elastic strain energy. Observing its failure, a desert sand-based backfill material specimen absorbs a large amount of energy under axial pressure and converts it into elastic properties. When the pressure it bore exceeded its yield limit, a fracture occurred at one end of the specimen first, and cracks occurred inside the specimen. The cracks gradually developed, increased, and penetrated the whole specimen, and some materials fell off, damaging the specimen. The failure results of numerical simulation are relatively close to the actual situation, so the numerical simulation can more accurately simulate the mechanical parameters and the failure mode of the specimen.

## 4. Analysis of Influencing Factors

The influence of sand content, porosity, desert sand size distribution, and model size on the compressive properties of the desert sand-based backfill material was studied by taking the values obtained from indoor results as basic calculation parameters.

### 4.1. Effect of Sand Content on Uniaxial Compressive Strength

**Figure 6:**
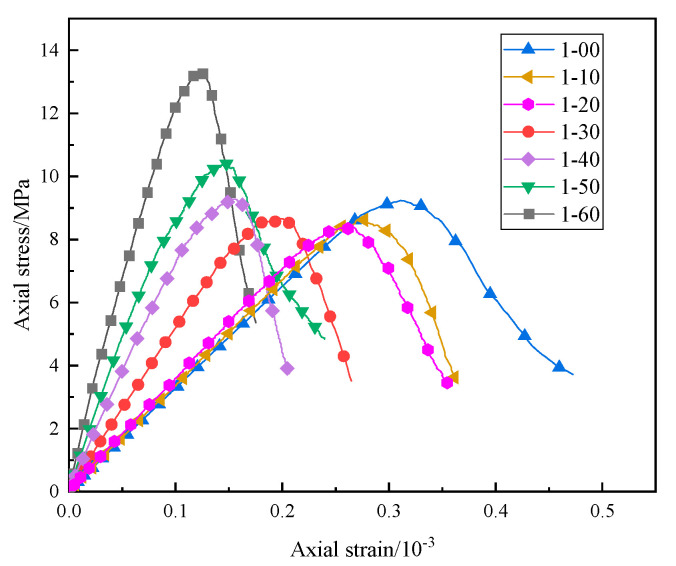
Stress–strain curve of uniaxial compression test for models with different sand content.

The sand content of the specimen was adjusted to 10%, 20%, 30%, 40%, 50%, and 60%, respectively; its uniaxial compression test was simulated, and a stress–strain curve was drawn, as shown in Figure 6. It can be seen that when the sand content is lower than 40%, the compressive performance of the specimen decreases slightly, while when the sand content is higher than 40%, the uniaxial compressive strength of the specimen has significantly improved, increasing to 13.3 MPa when the sand content is 60%.

Research on desert sand concrete has shown that the optimal sand content of desert sand is between 20 and 60%, and the numerical simulation results are approximately the same as the experimental results [30,35]. When the desert sand content is low, the compressive strength of the specimen decreases slightly. This is because when there is less desert sand, the specimen skeleton is restructured, and there are more large pores between the particles of high-water filling materials, which cannot be completely filled in the desert sand concrete. The average particle size of desert sand is small, and when the sand content is high, the tiny pores between aggregates can be filled in the production of filling materials, forming a dense skeleton and thus improving the compressive strength of the specimen. Unlike other tests, the indoor test in this study uses a high-water backfill material, not ordinary concrete. Material A of the HWBM is composed of sulfoaluminate cement and a suspension agent, while material B is generally composed of gypsum, lime, and an early strength agent. This material has a high slurry volume after adding water, enabling the desert sand to be better embedded in it. From the microstructure, it can also be seen that when high content of desert sand is added, the compactness of the desert sand-based backfill material is more compact [34]. Therefore, the high content of desert sand can effectively improve the compressive strength of desert sand-based backfill materials.

### 4.2. Effect of Porosity on Uniaxial Compressive Strength

**Figure 7:**
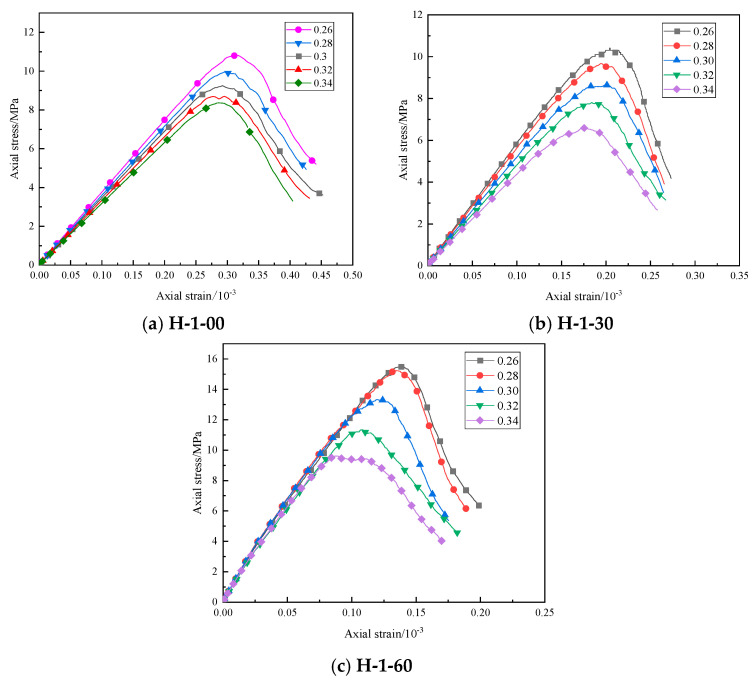
Stress–strain curve of the model uniaxial compression test under different porosity values.

The porosity was set at 0.26, 0.28, 0.30, 0.32, and 0.34. Figure 7 displays the uniaxial compression test stress–strain curves of models with different porosities. While other mesoscopic parameters remained unchanged, with the increase in porosity, the numerical simulation uniaxial compressive strength of different samples decreased gradually, and the peak strength also increased gradually as strain peaked. When the porosity increased from 0.3 to 0.34, the values of uniaxial compressive strength of specimens H-1-00, H-1-30, and H-1-60 decreased by 16.90%, 24.30%, and 28.01%, respectively.

The mesoscopic parameters of different specimen models under different porosity values are summarized in Table 4. With an increase in porosity, the number of particles in the model, the number of bonds between particles, the number of fractures, and the uniaxial compressive strength showed a downward trend. The number of particles contained in the model itself decreased, and so did the number of contact bonds. The contact bonding between particles corresponded to the microbonding surface or bonding point formed by the slurry cementation of the HWBM between desert sand aggregates. These microbonding surfaces and bonding points are important factors for maintaining the strength of desert sand-modified HWBM. Therefore, with the increase in porosity, the decrease in the number of contact bonds between particles was the main reason for the decrease in the uniaxial compressive strength. In addition, with the decrease in porosity, the particles are more closely arranged, and the intergranular embedding is stronger. Under the action of external forces, the position adjustment between particles is not easy to occur, and the resistance of particle clusters to deformation is enhanced, which shows that the compressive strength increases with the decrease in porosity.

### 4.3. Effect of Desert Sand Particle Size Distribution on Uniaxial Compressive Strength

**Figure 8:**
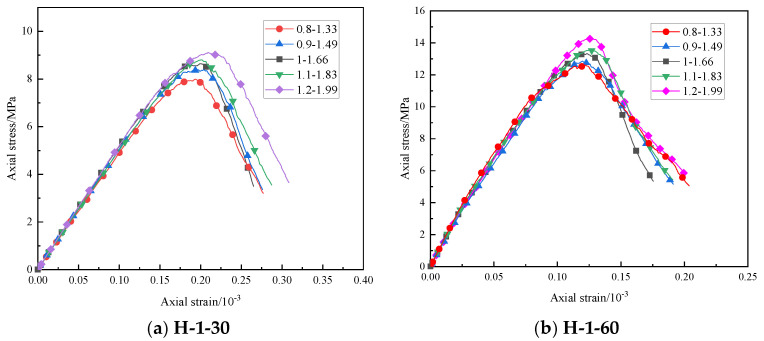
Stress–strain curve of the model uniaxial compression test under different desert sand particle size distributions.

Keeping other parameters unchanged, we changed the distribution range of desert sand particle size to 0.8~1.33 mm, 0.9~1.49 mm, 1~1.66 mm, 1.1~1.83 mm, and 1.2~1.99 mm and conducted a uniaxial compression simulation experiment. Figure 8 shows the stress–strain curve obtained from the experimental results. According to the results, the size distribution of desert sand had a great impact on the uniaxial compressive strength of the specimen. When the size distribution of desert sand increased from 0.8~1.33 mm to 1.2~1.99 mm, the uniaxial compressive strength of specimen H-1-30 increased from 7.99 MPa to 9.12 MPa, and the uniaxial compressive strength of specimen H-1-60 increased from 12.60 MPa to 14.29 MPa, increasing by 14.14% and 13.41%, respectively.

Therefore, increasing the size distribution of desert sand in a certain range can improve the compressive performance of desert sand-modified HWBMs. This is because when the other conditions are the same, the smaller the desert sand particles, the larger the specific surface area of desert sand per unit weight, and its water demand will also increase. Under the same amount of cementitious material, size distribution plays a role in reducing the strength of the specimen. However, when the particle size of desert sand increases, the specific surface area of desert sand per unit weight is larger, the particles of high-water backfill material in the contact area of particles are more, and the cohesive force is larger, which leads to the enhancement of the compressive strength of the specimen of desert sand-based backfill material. However, the strength of the specimen will be larger without blindly increasing the particle size of desert sand. To improve the compression resistance of desert sand-modified HWBM, the particle size distribution of desert sand can be improved within a certain range.

### 4.4. Effect of Model Size on Uniaxial Compressive Strength

The effect of model size on the specimen was studied by increasing the difference between 25 mm and 50 mm. The size effect degree is used to quantitatively analyze the size effect of compressive strength of desert sand-based filling material specimens [40]. Taking the 50 × 100 mm specimen as the benchmark specimen, the size effect degree *γ*_75_ and *γ*_100_ of the cylindrical desert sand-based backfill material specimen were defined as follows:

$$\gamma_{75}=\frac{f_{c,50}-f_{c,75}}{f_{c,50}}\times 100\%$$

$$\gamma_{100}=\frac{f_{c,50}-f_{c,100}}{f_{c,50}}\times 100\%$$

where *f_c_*_,50_, *f_c_*_,75_, and *f_c_*_,100_, respectively, represent the compressive strength of the sample with a model size of 50 × 100 mm, 75 × 150 mm, and 100 × 200 mm of the desert sand-based filling material.

**Figure 9:**
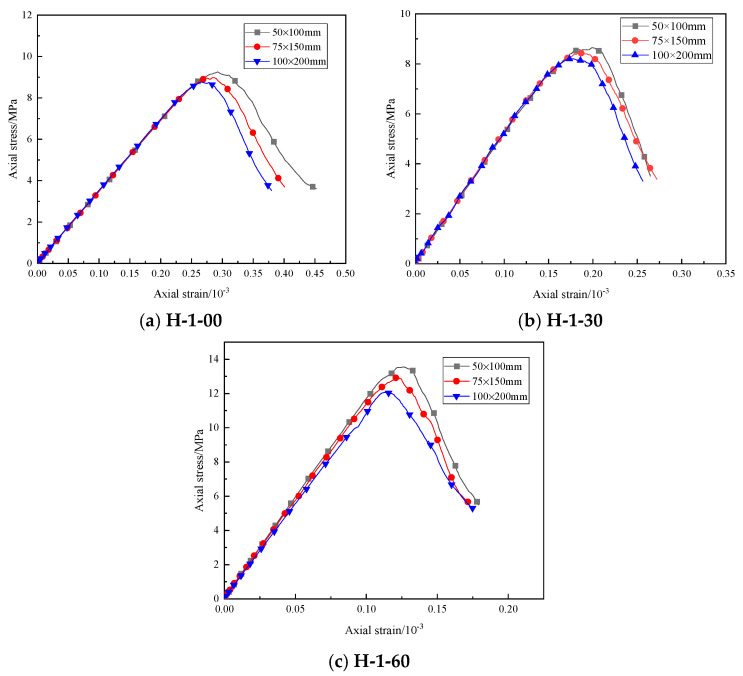
Stress–strain curves of uniaxial compression tests for models of different sizes.

Figure 9 shows the stress-strain curves of specimens with different model sizes. It can be seen that with the increase in the model size, the uniaxial compressive strength of the specimen decreased. Detailed parameters are shown in Table 5. When the model size was from 50 × 100 mm to 100 × 200 mm, the uniaxial compressive strength of specimen H-1-00 decreased from 9.24 MPa to 8.75 MPa, the uniaxial compressive strength of specimen H-1-30 decreased from 8.68 MPa to 8.20 MPa, and the uniaxial compressive strength of specimen H-1-60 decreased from 13.33 MPa to 12.03 MPa. According to the calculation, when the model size is 75 × 150 mm, the size effect degree of the three specimens is 2.92%, 3.00%, and 3.60%, respectively. When the model size is 100 × 200 mm, the size effect degree of the three specimens is 5.30%, 5.53%, and 9.75%, respectively.

According to the size effect theory of Bazant [41] fracture mechanics, the instability and expansion of fracture zones cause the failure of quasi-brittle materials such as concrete, and the development of fracture zones formed by micro-cracks is related to energy consumption and the release of stored energy. There is a fracture process zone composed of micro-cracks in the front end of concrete cracks. The expansion of cracks in the fracture process zone involves the release and consumption of energy. Concrete fracture is caused by the mismatch between the release and consumption of stored energy in size.

Bazant adopted the method of dimensional analysis, combined with experiments, and made use of energy balance to deduce the formula of size effect, as shown below.

$$\sigma_{N}=\frac{Bf_{t}}{\sqrt{1+\beta }},\beta =\frac{D}{D_{0}}$$

where *f_t_*, *B*, and *D*, respectively, represent the tensile strength, infinite programmatic number, and constant dependent on the structural geometry of the material.

It can be seen that the compressive strength of the specimen decreases with the increase in size. The compressive strength of desert sand-based backfill material specimens exhibits a relatively obvious size effect. However, the increased size of desert sand-based backfill material still has good compressive performance.

## 5. Conclusions

In this paper, through a series of experiments, the effects of water–cement ratio and desert sand doping amount on the properties of desert sand-based backfill material were studied. At the same time, based on the discrete element particle flow software PFC3D, the three-dimensional structure of the desert sand-based backfill material model was established, and the influence of sand content, porosity, desert sand particle size distribution, and model size on the performance of the specimen was analyzed. Conclusions are summarized as follows:

(1) The initial setting time of the high-water filling material modified by desert sand is reduced; the compressive strength and elastic modulus of the high-water filling material can be effectively improved by the high-water sand mixed with high content, and the compressive strength of the specimen decreases with the increase in the water–cement ratio;
(2) With the increase in desert sand content, the compressive strength of desert sand-based filling materials decreases first and then increases, and all specimens with sand content between 40 and 60% have good compressive performance;
(3) The uniaxial compressive strength of numerical simulation is related to the particle size distribution of desert sand. In a certain range, increasing the particle size distribution of desert sand can improve the compressive performance of desert sand-based backfill material specimens;
(4) The specimen size effect of desert sand-based backfill material is significant, and its compressive performance decreases with the increase in model size.

The discrete element particle flow software PFC3D can simulate and predict the mechanical properties and failure process of desert sand-based backfill materials. Due to the problems of the PFC3D software and algorithm, the calculation efficiency is not high, which greatly limits the calculation scale. The desert sand-based backfill material contains two kinds of particles with different sizes and properties, namely desert sand and high-water backfill material. In order to simplify the complexity of the problem and improve the computational efficiency, the effect of gradation effect was not considered in this paper. Therefore, it is necessary to consider the effect of gradation effect on the macro and micro mechanical properties of desert sand-based backfill materials in the future, and carry out in-depth research.

## Figures and Tables

**Table 1:** Specific parameters of test pieces.

Specimen	W/C	Sc	Width (mm)	Height (mm)
H-1-00	1	0%	50.20	100.40
H-1-30	1	30%	50.10	100.60
H-1-60	1	60%	50.50	101.20
H-1.5-00	1.5	0%	50.30	100.20
H-1.5-60	1.5	60%	50.20	100.50
H-2-00	2	0%	50.60	100.80
H-2-60	2	60%	50.10	100.40

**Table 2:** Micromechanical parameters of the numerical model.

Specimen	emod/Pa	kratio	pb_kratio	pb_emod/Pa	pb_ten/Pa	pb_coh/Pa	fric
HWBM	18 × 10^9^	1.0	1.0	18 × 10^9^	4.0 × 10^6^	3.0 × 10^6^	0.35
Sand	200 × 10^9^	1.0	1.0	200 × 10^9^	25.0 × 10^6^	21.0 × 10^6^	0.55

**Table 3:** Mechanical parameters and failure modes of the laboratory test and the numerical simulation.

Variable	Indoor Test		Numerical Simulation		Specimen
	Mechanical Parameters	Failure Form	Failure Form	Mechanical Parameters	
Peak intensity/MPa	9.20	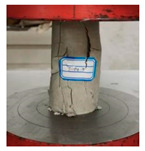	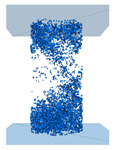	9.24	H-1-00
Elastic modulus/GPa	0.57			0.56	
Peak strain/10^−3^	0.30			0.29	
Peak intensity/MPa	9.04	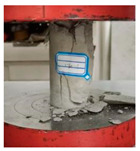	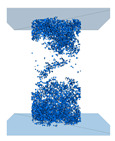	9.09	H-1-30
Elastic modulus/GPa	0.82			0.78	
Peak strain/10^−3^	0.20			0.21	
Peak intensity/MPa	13.52	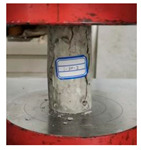	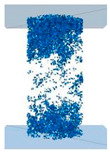	13.55	H-1-60
Elastic modulus/GPa	1.28			1.19	
Peak strain/10^−3^	0.12			0.13	

**Table 4:** Macroscopic parameters and compressive strength of the numerical model.

Specimen	Porosity	Balls	Contacts	Fractures	*f_c_*/MPa
H-1-00	0.26	3302	11,957	5602	10.80
	0.28	3217	11,435	5333	9.94
	0.30	3129	10,925	4948	9.24
	0.32	3041	10,333	4635	8.68
	0.34	2956	9650	4420	8.35
H-1-30	0.26	5804	24,044	12,470	10.30
	0.28	5622	22,522	11,703	9.67
	0.30	5436	20,633	10,426	8.68
	0.32	5258	19,113	8735	7.79
	0.34	5137	17,442	7598	6.57
H-1-60	0.26	9814	42,359	10,800	15.47
	0.28	9478	39,403	10,394	15.23
	0.30	9226	37,230	9566	13.33
	0.32	8974	34,786	8296	11.33
	0.34	8720	31,631	8248	9.59

**Table 5:** Uniaxial test results of models of different sizes.

Specimen	Size/mm (Width × Height)	*f_c_*/MPa	*ε_u_*/10^−3^
H-1-00	50 × 100	9.24	0.29
	75 × 150	8.97	0.27
	100 × 200	8.75	0.26
H-1-30	50 × 100	8.68	0.20
	75 × 150	8.42	0.18
	100 × 200	8.20	0.17
H-1-60	50 × 100	13.33	0.13
	75 × 150	12.85	0.12
	100 × 200	12.03	0.11

## Data Availability

The research data used to support the findings of this study are currently under embargo while the research findings are commercialized. Requests for data 12 months after the publication of this article will be considered by the corresponding author.
